# Characterization and immunogenicity of nanoparticle vaccines displaying embecovirus spike proteins

**DOI:** 10.1002/btpr.88530

**Published:** 2026-06-15

**Authors:** Peter J. Halfmann, Augustine Duffy, Alex Wee, Noah Anderson, Jackson Terrell, Yoshihiro Kawaoka, Ravi S. Kane

**Affiliations:** ^1^ Department of Pathobiological Sciences, School of Veterinary Medicine Influenza Research Institute, University of Wisconsin Madison Wisconsin USA; ^2^ School of Chemical & Biomolecular Engineering Georgia Institute of Technology Atlanta Georgia USA; ^3^ Wallace H. Coulter Department of Biomedical Engineering Georgia Institute of Technology Atlanta Georgia USA; ^4^ Division of Virology, Department of Microbiology and Immunology Institute of Medical Science, University of Tokyo Tokyo Japan; ^5^ The Research Center for Global Viral Diseases National Center for Global Health and Medicine Research Institute Tokyo Japan; ^6^ Pandemic Preparedness, Infection and Advanced Research Center (UTOPIA) University of Tokyo Tokyo Japan

**Keywords:** common cold, coronavirus, embecovirus, nanoparticle, spike protein, vaccine

## Abstract

Endemic human coronaviruses OC43 and HKU1 cause widespread respiratory infections and can be associated with severe illness in immunocompromised and elderly individuals. Frequent adaptive evolution in the spike proteins of these embecoviruses and the potential for zoonotic transmission from a large animal reservoir necessitates the characterization of the immunogenic landscape of the spike proteins of embecoviruses. Here, we constructed nanoparticle vaccines displaying the spike antigens from OC43, HKU1 A, or HKU1 B, as well as a bivalent formulation incorporating spike antigens from OC43 and HKU1 A. Immunization of mice elicited spike‐specific IgG antibody responses, with endpoint titers demonstrating cross‐reactivity among clade‐matched viruses. Notably, the bivalent formulation elicited antibody responses comparable to those of monovalent vaccines against matched antigens. These findings inform future design of vaccines against human‐infecting embecoviruses and could serve as an important step toward a universal vaccine against common cold causing coronaviruses.

## Graphical abstract

Virus‐like particle vaccines displaying spike proteins from OC43, HKU1 A, and HKU1 B were evaluated in mice for their ability to elicit antibodies against the spike proteins from OC43, HKU1 A, HKU1 B, and HKU1 C.

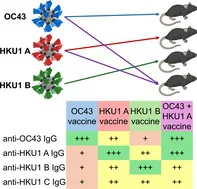

## 1 INTRODUCTION

Within the past 26 years, coronaviruses have been gaining attention due to human outbreaks caused by multiple viruses within the family, including severe acute respiratory syndrome (SARS‐CoV) in 2002,^1^ Middle East respiratory syndrome (MERS‐CoV) in 2012,^2^ and severe acute respiratory syndrome 2 (SARS‐CoV‐2) in 2019.^3^ In parallel, several low‐pathogenic coronaviruses—OC43, HKU1, 229E, and NL63^4^—circulate endemically in humans and are responsible for a significant fraction of common cold infections. Of these, OC43 and HKU1 both belong to the *Embecovirus* subgenus of *Betacoronavirus*. Both viruses have been linked to severe respiratory tract infections in elderly and immunocompromised individuals, pneumonia,^5^ and encephalitis in infants.^6^ Peak circulation of these viruses occurs in winter months, with OC43 frequently detected in both children and adults.^7^ Beyond endemic seasonal transmission in humans, embecoviruses also circulate widely in animal hosts. Equine coronavirus,^8^ porcine hemagglutinating encephalomyelitis virus,^9^ canine respiratory coronavirus,^10^ and bovine coronavirus^11^ collectively comprise a large reservoir of viruses circulating in animal hosts that presents ongoing opportunities for zoonotic transmission to humans. Notably, transmission of bovine coronavirus to humans has already been documented.^12^

Like other coronaviruses, OC43 and HKU1 utilize a spike (S) protein to facilitate viral entry into a host cell.^13^, ^14^ The N‐terminal domains (NTDs) of both HKU1 and OC43 S proteins bind to 9‐O‐acetylated sialic acid as an initial host‐cell ligand; notably, this sialic‐acid binding pocket is conserved between OC43 and HKU1.^15^ This NTD binding is necessary for infection as it has been shown to lead to the opening of the RBD and the engagement of TMPRSS2 as an entry receptor^16^, ^17^, ^18^ in HKU1. The conserved NTD binding pocket between HKU1 and OC43 presents the possibility of eliciting cross‐neutralizing antibodies against both S proteins in a single vaccine. The study presented here aims to examine the immunogenicity of full embecovirus S antigens and the cross‐reactivity of the elicited antibodies against select embecovirus strains.

The current body of work surrounding vaccines targeting OC43 and HKU1 remains limited. While structural information^19^, ^20^ and binding sites^13^ have been described, the immunogenic properties and broader antigenic landscape of OC43 and HKU1 S have not been explored as extensively as for sarbecovirus and merbecovirus antigens.^21^, ^22^, ^23^, ^24^ Although a recent study using an mRNA vaccine platform demonstrated the protective efficacy of mRNA‐OC43 in mice against OC43 and murine hepatitis virus (MHV‐A59),^25^ studies examining whether nanoparticle‐based vaccines displaying human‐infecting embecoviruses can elicit antibody responses across the full *Embecovirus* subgenus remain limited. This knowledge gap is particularly consequential, as both OC43 and HKU1 are capable of seasonal reinfection at appreciable rates,^26^ and the OC43 S protein has also been shown to continuously evolve in the S1 region as a result of selective pressures.^27^

The high rate of viral adaptation, together with the potential for zoonotic spillover, underscores an unmet need to better characterize how immune recognition of the embecovirus S protein varies across strains. We expressed and purified OC43 and HKU1 S proteins for multivalent display on virus‐like particles (VLPs) and examined their immunogenicity in mice. Sera from mice immunized with these vaccines exhibited some limited cross‐reactivity across species but with the highest endpoint IgG titers against the homologous antigens.

## 2 MATERIALS AND METHODS

### 2.1 Expression and purification of S proteins

Plasmids encoding the S proteins of HCoVs OC43/Canada/2021 (accession WAK98266.1), OC43/China/07/2017 (OC43 J; accession QRK03812.1), HKU1 A/China/2018 (accession URC25127.1), HKU1 B/Japan/2020 (accession BDB58106.1), and HKU1 C/Hong Kong/2006 (accession ABD75625.1) each with an AviTag, 2P mutations,^28^ a Foldon trimerization domain, and a hexahistidine tag (Table S1) were synthesized by Gene Universal (Newark, DE) and cloned into pcDNA3.1 (−). Spike proteins were then expressed in Expi293F cells, purified via immobilized metal affinity chromatography (IMAC) and size exclusion chromatography, and enzymatically biotinylated as described previously.^29^, ^30^, ^31^

### 2.2 Assembly of VLP‐S

MS2 and streptavidin (SA) were expressed and purified in *E. coli* as previously described,^21^, ^22^, ^23^, ^29^, ^30^, ^31^, ^32^, ^33^, ^34^, ^35^, ^36^, ^37^ and MS2‐SA VLPs were then assembled and purified as previously described.^21^, ^22^, ^23^, ^29^, ^30^, ^31^, ^34^, ^35^, ^36^, ^37^ In brief, MS2 was purified from *E. coli* lysate via Capto Core 700 (Cytiva) and size exclusion chromatography (SEC) and then biotinylated enzymatically, and SA was purified from *E. coli* inclusion bodies. MS2‐SA was then assembled by the slow addition of MS2 to a 20‐fold molar excess of SA, and excess SA was removed by SEC. VLP‐S was assembled by mixing MS2‐SA with biotinylated spike protein at a ratio at which no unbound spike protein was detected via analytical SEC.

### 2.3 In vitro characterization of VLP‐S

Purity, hydrodynamic radius, and proper antigenic presentation of VLP‐S were characterized as described previously^21^, ^22^, ^23^, ^29^, ^30^, ^31^, ^32^, ^33^, ^34^ by sodium dodecyl sulfate‐polyacrylamide gel electrophoresis (SDS‐PAGE), dynamic light scattering (DLS), and enzyme‐linked immunosorbent assay (ELISA), respectively.

### 2.4 Ethics approval and animal immunizations

Animal studies were performed at the University of Wisconsin‐Madison under an approved protocol (V006426). Animals were housed in a temperature‐controlled and humidity‐controlled environment with 12 h light and dark cycles. Prior to the start of a study, animals were allowed to acclimate to the animal room for at least three days. Enrichment was provided, and water and food were provided ad libitum.

C57BL/6 mice were purchased from Jackson Laboratories. All mice were female and at the age of 10–12 weeks at the start of the studies. Mice were immunized by subcutaneous injection with a total volume of 250 μL of an equal volume mixture of VLP vaccine preparations (7.5 μg of each S antigen) and Alhydrogel adjuvant. For serum collection, blood was obtained via terminal cardiac puncture while mice were under deep isoflurane anesthesia.

### 2.5 Serum endpoint titers

Endpoint titers were determined by conducting a single assay using sera from each mouse (*n* = 5). The data is presented as geometric mean ± geometric SD; significance was determined by Brown–Forsythe and Welch ANOVA and Games–Howell's multiple comparisons between groups for OC43 J, OC43 Canada, HKU1 A, HKU1 B, and HKU1 C proteins.

## 3 RESULTS AND DISCUSSION

### 3.1 Selection of virus strains for immunizations

Antigens from recent strains were selected to span both OC43 and HKU1 lineages on the phylogenetic tree (Figure 1). OC43/Canada/2021 was chosen as the representative OC43 vaccination strain due to its status as a recently isolated strain. Additionally, this OC43 strain lies on the main branch within the OC43 phylogeny, suggesting that it shares conserved antigenic features with a large fraction of circulating strains and may therefore be well suited to elicit cross‐reactive antibodies within the clade. HKU1 is phylogenetically split into three genotypes, A, B, and C,^38^ necessitating multiple representative strains. HKU1 A/China/2018 and HKU1 B/Japan/2020 were selected as representative strains of the HKU1 A and HKU1 B genotypes, respectively, using the same phylogenetic selection strategy applied to OC43, with the aim of promoting cross‐reactive antibody responses within the HKU1 clade. We hypothesized that antibody responses against HKU1 C could be elicited through the presentation of the HKU1 B S protein, since the C genotype is a recombination of A and B.^38^ As such, a representative strain for HKU1 C was not chosen.

**FIGURE 1:**
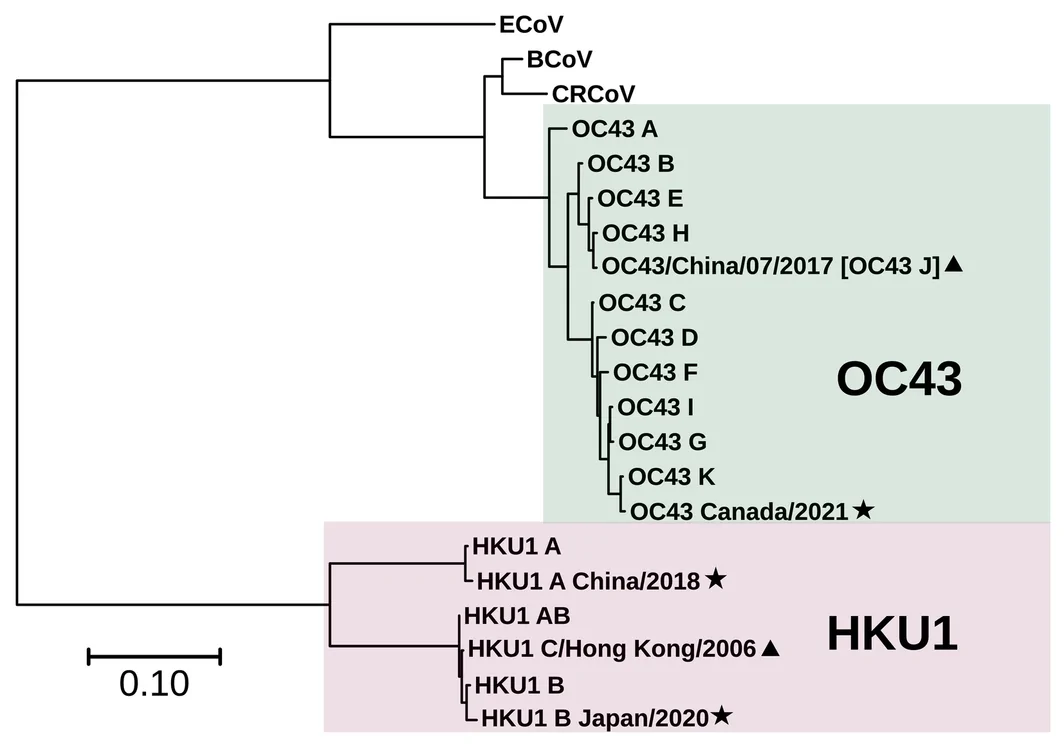
Amino acid phylogenetic tree of selected Embecovirus S proteins. The scale bar represents the number of mutations per residue. Animal‐infecting embecoviruses shown here include equine coronavirus (ECoV), bovine coronavirus (BCoV), and canine respiratory coronavirus (CRCoV). Embecoviruses highlighted by the green rectangle are OC43 viruses, while those highlighted by the purple rectangle are HKU1 viruses. Embecovirus strains used for vaccines are marked by a **★**. Embecovirus strains used to supplement enzyme‐linked immunosorbent assay (ELISA) endpoint studies in addition to vaccine strains are marked by a ▲.

### 3.2 Generation and characterization of VLP‐S

We previously developed a SA‐coated MS2 nanoparticle construct.^23^, ^29^, ^30^, ^34^, ^35^, ^36^ An AviTag inserted into the surface loop of an MS2 coat protein dimer enables site‐specific coating of SA, which enables the presentation of diverse viral antigens.^22^, ^23^, ^29^, ^30^, ^37^ Both MS2 coat protein dimers and SA were expressed in BL21(DE3) *Escherichia coli* and purified by size exclusion chromatography. The resulting MS2 was biotinylated and added dropwise to an excess of SA to construct MS2‐SA particles. Unbound SA was removed via SEC.

The selected S protein antigens were each modified to include two stabilizing proline (2P) mutations^24^ as well as a C‐terminal foldon trimerization domain, AviTag, and hexahistidine tag. Plasmids encoding the modified proteins were transfected into Expi293F mammalian cells, and the resulting proteins were subsequently purified. S proteins were then biotinylated using the biotin ligase BirA and mixed with an appropriate ratio of MS2‐SA to produce VLP‐S (Figure 2a). The appropriate ratio of MS2‐SA to S protein antigen was defined as the minimum amount of MS2‐SA that did not result in an excess S protein peak on an SEC chromatogram.

**FIGURE 2:**
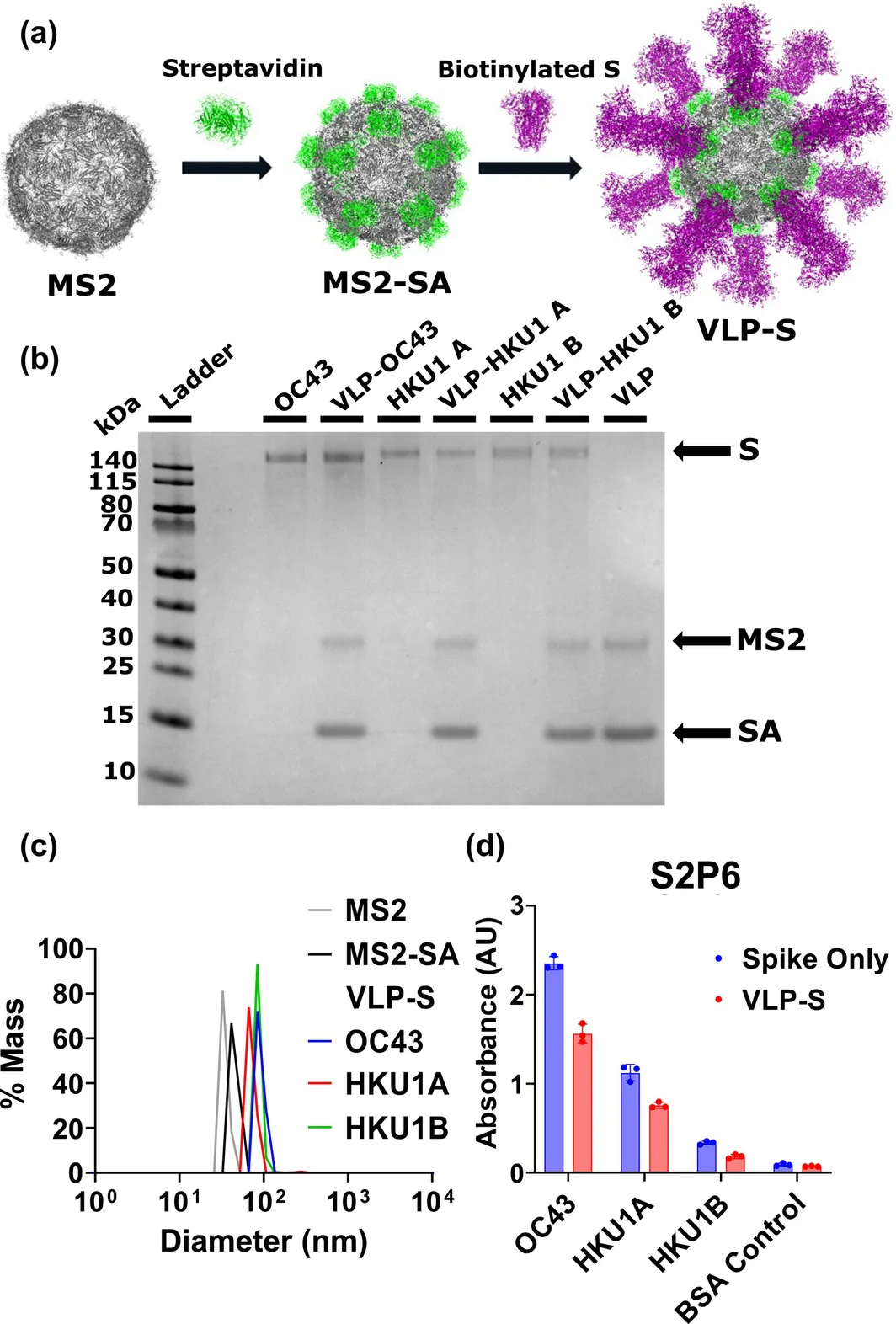
(a) Schematic of VLP‐S construction. (MS2 [gray], PDB: 2MS2; SA [green], PDB: 3RY2; S [purple], PDB: 6VSB). (b) Characterization of S protein antigens and VLP‐S by sodium dodecyl sulfate‐polyacrylamide gel electrophoresis (SDS‐PAGE). An uncropped gel is available in Figure S1. (c) Characterization of MS2, MS2‐SA, VLP‐OC43, VLP‐HKU1 A, and VLP‐HKU1 B by dynamic light scattering. Measurements are reported as % mass using the isotropic spheres model. (d) Characterization of recognition of embecovirus S antigens and VLP‐S by the S2‐binding monoclonal antibody S2P6 (63 ng/mL) using an enzyme‐linked immunosorbent assay. Each data point represents one technical replicate (*n* = 3), and each bar represents the arithmetic mean with standard deviation. A concentration dependent enzyme‐linked immunosorbent assay (ELISA) is available in Figure S2.

The purified antigen and resulting VLP‐S were characterized to ensure purity and successful assembly. SDS‐PAGE analysis of each constructed VLP‐S showed purity of the S and VLP‐S (Figure 2b). MS2 appeared at approximately 30 kDa, and SA appeared at approximately 15 kDa, matching known molecular weights. S proteins appeared larger than 140 kDa due to their extensive glycosylation. DLS measurements of each VLP‐S indicate hydrodynamic diameters between 90 and 100 nm, matching expected values for an S protein on MS2‐SA^29^ compared to diameters of 46 nm for MS2‐SA and 36 nm for MS2 alone (Figure 2c). An ELISA was performed with purified S proteins and their respective VLP‐S against a broadly cross‐reactive S2‐binding monoclonal antibody, S2P6^39^ (Figure 2d, Figure S2). The binding activity of OC43 and HKU1 A S and VLP‐S follows known trends^39^ with OC43 showing robust binding while HKU1 A displays weaker binding. HKU1 B displayed weaker binding than either OC43 or HKU1 A but still displayed some recognition by S2P6.

### 3.3 VLP‐S elicits cross‐reactive antibodies against embecoviruses in mice

Next, we examined the immunogenicity of each VLP‐S vaccine in mice. Groups of five female C57BL/6 mice were immunized subcutaneously with a single dose of VLP‐OC43 S, VLP‐HKU1A S, VLP‐HKU1B S (7.5 μg of the S protein), or a corresponding VLP‐only control. A fifth group of mice was immunized with a vaccine consisting of a mixture of VLP‐OC43 S and VLP‐HKU1A S (7.5 μg of each S protein antigen) to determine whether a cocktail elicited a more cross‐reactive antibody response against embecoviruses than single VLP‐S vaccines. All vaccines were adjuvanted with Alhydrogel. Three weeks after immunization, the mice were bled and the collected sera were tested against a panel of OC43 and HKU1 S proteins via ELISA (Figure 3a–e).

**FIGURE 3:**
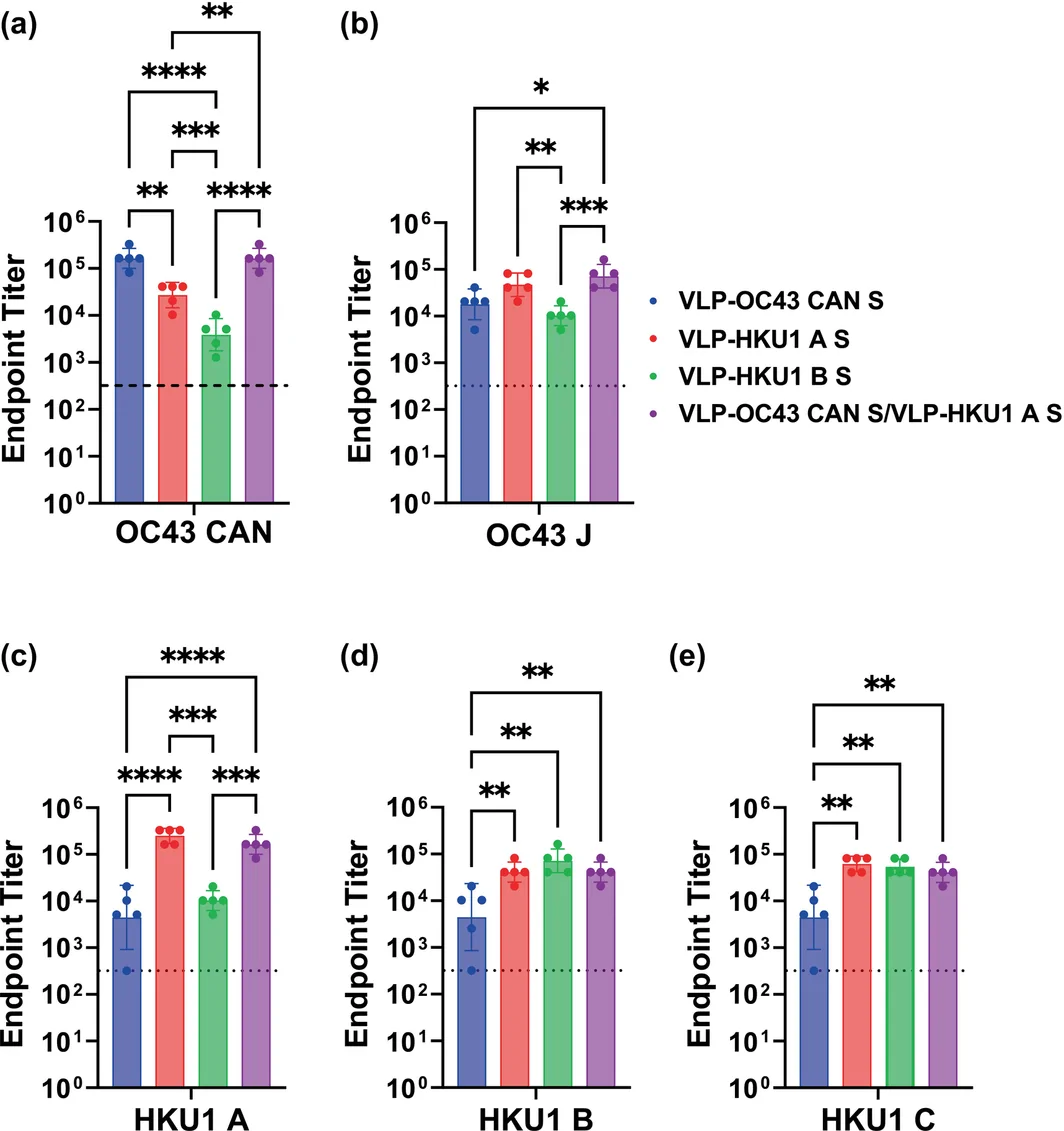
IgG endpoint titers against S protein of: (a) HCoV OC43/Canada/2021 (OC43) (b) OC43/China/07/2017 (OC43 J) (c) HKU1 A/China/2018 (HKU1 A) (d) HKU1 B/Japan/2020 (HKU1 B) (e) HKU1 C/Hong Kong/2006 (HKU1 C). ● indicate data from individual mice (geometric mean with geometric SD, *n* = 5: sera from 5 mice). ****p <0.0001, ***p <0.001, **p <0.01, *p <0.05, not significant comparisons are unlabeled. Significance was determined by lognormal one‐way ordinary ANOVA and Tukey post hoc multiple comparisons between groups. Detection limit (dashed line) = 320.

As expected, all VLP‐S vaccine groups exhibited high IgG antibody endpoint titers against their matched antigens. The bivalent vaccine elicited endpoint titers against each matched antigen that were statistically indistinguishable from those elicited by the corresponding monovalent vaccines, demonstrating that inclusion of multiple antigens does not diminish antigen‐specific immunogenicity for our VLP construct. Mice immunized with the OC43/HKU1 A cocktail group showed significantly higher antibody endpoint titers against OC43 J than the OC43 only group (Figure 3b; *p* <0.05). This amplified immune response likely occurs due to conserved epitopes between HKU1 A and OC43, which results in improved immunogenicity of the cocktail vaccine incorporating both antigens as compared to the monovalent VLP‐OC43 S vaccine. Notably, endpoint titers were comparable across HKU1 variants for each HKU1 antigen tested, with the sole exception that HKU1 A elicited significantly higher endpoint titers than HKU1 B when tested against the HKU1 A antigen (Figure 3c; *p* <0.001); no significant differences were observed in the reciprocal or heterologous comparisons (Figure 3c–e).

## 4 CONCLUSION

In this study we designed and generated VLP vaccines multivalently displaying the S proteins of OC43, HKU1 A, and HKU1 B. Vaccination of mice with VLP‐S resulted in high IgG endpoint titers against antigenically matched strains as is expected from our previous work.^21^, ^23^, ^35^, ^37^ A cocktail vaccine consisting of equal parts of VLP‐S displaying S from OC43 and HCoV‐HKU1 A achieved endpoint titers comparable to or higher than matched vaccines. Additionally, the cocktail vaccine elicited significantly higher endpoint titers against a heterologous strain of OC43 than the monovalent OC43 VLP‐S. There are likely to be conserved epitopes between OC43 and HKU1 A such as the sialic acid‐binding pocket in the NTD,^13^ and as a result a cocktail containing both boosts the immune response. Future studies optimizing mixture vaccines, possibly by increasing the number of formulation species or by adjusting strain selection, may be of value. Furthermore, our findings indicate that cocktail vaccines against embecoviruses may only require a single HKU1 strain to generate high IgG antibody endpoint titers against the entire HKU1 clade, leading to more effective vaccine formulations relying on fewer antigens. A prior study developed an OC43 mRNA vaccine and tested the protective efficacy of the vaccine in mice against a neurovirulent OC43 strain and MHV.^25^ While the findings highlight OC43‐specific antibody elicitation and cross‐reactivity to a related animal embecovirus, no cross‐reactivity was tested against other human‐infecting coronaviruses. Our findings further define the antigenic landscape of embecovirus S protein antigens by demonstrating clear cross‐reactivity among human strains, spanning both inter‐clade and intra‐subclade relationships.

Characterization of neutralizing antibodies elicited by our vaccines was a limitation of this study. We could not procure a suitable challenge virus for OC43‐directed neutralization studies, and no mouse challenge models currently exist for HKU1.^40^ Further characterization including challenge studies may provide valuable insight into the in vivo efficacy of the embecovirus S protein antigens in vaccines. Nevertheless, our work highlights a potential path toward cross‐reactive vaccines against embecoviruses and the immunogenic properties of their S proteins displayed on nanoparticles.

## AUTHOR CONTRIBUTIONS

**Peter J. Halfmann:** Conceptualization; investigation; data curation; resources; formal analysis; writing – original draft; visualization. **Noah Anderson:** Investigation; resources. **Augustine Duffy:** Conceptualization; investigation; data curation; resources; formal analysis; writing – original draft. **Yoshihiro Kawaoka:** Supervision; funding acquisition; project administration; writing – review and editing. **Jackson Terrell:** Investigation; resources. **Ravi S. Kane:** Conceptualization; supervision; funding acquisition; project administration; writing – original draft. **Alex Wee:** Investigation; resources; data curation; formal analysis; writing – original draft; visualization.

## CONFLICT OF INTEREST STATEMENT

The authors declare no competing interest.

## Supporting information


**Figure S1.** Unprocessed image of SDS‐PAGE gel displayed in Figure 2b.
**Figure S2.** Concentration‐dependent ELISA of S2P6 against OC43 S, HKU1 A S, and HKU1 B S (*n* = 3 technical replicates, arithmetic mean ± standard deviation).
**Table S1.** Protein sequences.

## Data Availability

The data supporting the conclusions of this work are available in the manuscript and Supporting Information.
