# Supplementary material for: Characterization and immunogenicity of nanoparticle vaccines displaying embecovirus spike proteins
Source: Biotechnol Prog. 2026 Jun 15;42(5):e88530. doi: 10.1002/btpr.88530 (PMC13636613; doi:10.1002/btpr.88530)
Supplement: Supplementary file 1 [file BTPR-42-e88530-s001.docx]

**Characterization and Immunogenicity of Nanoparticle Vaccines Displaying Embecovirus Spike Proteins**

Peter J. Halfmann^1, †^, Augustine Duffy^2, †^, Alex Wee^2, †^, Noah Anderson^3^, Jackson Terrell^2^, Yoshihiro Kawaoka^1,4,5,6^*, Ravi S. Kane^2,3^*

^1^Influenza Research Institute, Department of Pathobiological Sciences, School of Veterinary Medicine, University of Wisconsin, Madison, WI, 53711, USA

^2^School of Chemical & Biomolecular Engineering, Georgia Institute of Technology, Atlanta, Georgia, 30332, USA

^3^Wallace H. Coulter Department of Biomedical Engineering, Georgia Institute of Technology, Atlanta, Georgia, 30332, USA

^4^Division of Virology, Department of Microbiology and Immunology, Institute of Medical Science, University of Tokyo, Tokyo 108-8639, Japan

^5^The Research Center for Global Viral Diseases, National Center for Global Health and Medicine Research Institute, Tokyo 162-8655, Japan.

^6^Pandemic Preparedness, Infection and Advanced Research Center (UTOPIA), University of Tokyo, Tokyo 162-8655, Japan

^†^ These authors contributed equally

*To whom correspondence should be addressed:
yoshihiro.kawaoka@wisc.edu, ravi.kane@chbe.gatech.edu

**Supporting Figure 1.** Unprocessed image of SDS-PAGE gel displayed in Figure 2b.

**Supporting Figure 2:** Concentration-dependent ELISA of S2P6 against OC43 S, HKU1 A S, and HKU1 B S (n = 3 technical replicates, arithmetic mean ± standard deviation).

**Supporting Table 1:** Protein sequences

| **Protein Name** | **Amino Acid Sequence** |
| --- | --- |
| MS2-AviTag | MASNFTQFVLVDNGGTGDVTVAPSNFANGVAEWISSNSRSQAYKVTCSVRQSSAQNRKYTIKVEVPKVATQTVGGVELPVAAWRSYLNMELTIPIFATNSDCELIVKAMQGLLKDGNPIPSAIAANSGIYASNFTQFVLVDNGGGLNDIFEAQKIEWHETGDVTVAPSNFANGVAEWISSNSRSQAYKVTCSVRQSSAQNRKYTIKVEVPKVATQTVGGVELPVAAWRSYLNMELTIPIFATNSDCELIVKAMQGLLKDGNPIPSAIAANSGIY |
| OC43/Canada/2021 S protein | MFLILLISLPTAFAVIGDLNCTLDPRLKGSFNNRDTGPPSISIDTVDVTNGLGTYYVLDRVYLNTTLFLNGYYPTSGSTYRNMALKGTDLLSTLWFKPPFLSDFINGIFAKVKNTKVFKDGVMYSEFPAITIGSTFVNTSYSVVVQPRTINSTQDGVNKLQGLLEVSVCQYNMCEYPHTICHPNLGNHFKELWHYDTGAVSCLYKRNFTYDVNATYLYFHFYQEGGTFYAYFTDTGFVTKFLFNVYLGMALSHYYVMPLTCIRRSKDGFNPEYWVTPLTPRQYLLAFNQDGIIFNAVDCMSDFMSEIKCKTQSIAPPTGVYELNGYTVQPIADVYRRKPDLPNCNIEAWLNDKSVPSPLNWERKTFSNCNFNMSSLMSFIQADSFTCNNIDAAKIYGMCFSSITIDKFAIPNRRKVDLQLGNLGYLQSSNYRIDTTATSCQLYYNLPAANVSVSRFNPSTWNKRFGFIEDSVFKPQPAGVLTDHDVVYAQHCFKAPKNFCPCSSCSGKNNGIGTCPAGTNSLTCDNLCTLDPITLKAPDTYKCPQSKSLVGIGEHCSGLAVKSDYCGNNSCTCQPQAFLGWSADSCLQGDKCNIFANFILHDVNNGLTCSTDLQKANTEIELGVCVNYDLYGISGQGIFVEVNATYYNSWQNLLYDSNGNLYGFRDYITNRTFMIHSCYSGRVSAAYHANSSEPALLFRNIKCNYVFNNSLTRQLQPINYSFDSYLGCVVNAYNSTAISVQTCDLTVGSGYCVDYFKNRGSASAITTGYRFTNFEPFTVNSVNDSLEPVGGLYEIQIPSEFTIGNMEEFIQTSSPKVTIDCAAFVCGDYAACKLQLVEYGSFCDNINAILTEVNELLDTTQLQVANSLMNGVTLSTKLKDGVNFNVDDINFAPVLGCLGSECSKASSRSAIEDLLFDKVKLSDVGFVEAYNNCTGGAEIRDLICVQSYKGIKVLPPLLSENQISGYTLAATSASLFPPWTAAAGVPFYLNVQYRINGLGVTMDVLSQNQKLIANAFNNALHAIQQGFDATNSALVKIQAVVNANSEALNNLLQQLSNRFGAISASLQEILSRLDPPEAEAQIDRLINGRLTALNAYVSQQLSDSTLVKFSAAQAMEKVNECVKSQSSRINFCGNGNHIISLVQNAPYGLYFIHFNYVPTKYVTAKVSPGLCIAGNRGIAPKSGYFVNVNNTWMYTGSGYYYPEPITENNVVVMSTCAVNYTKAPYVMLNTSIPNLPDFKEELDQWFKNQTSVAPDLSLDYINVTFLDLQVEMNRLQEAIKVLNHSYINLKDIGTYEYGSGYIPEAPRDGQAYVRKDGEWVLLSTFLGGLNDIFEAQKIEWHEHHHHHH |
| OC43 J S protein | MFLILLISLPTAFAVIGDLNCTLDPRLKGSFNNRDTGPPSISIDTVDVTNGLGTYYVLDRVYLNTTLFLNGYYPTSGSTYRNMALKGTDLLSTLWFKPPFLSDFINGIFAKVKNTKVFKDGVMYSEFPAITIGSTFVNTSYSVVVQPRTINSTQDGVNKLQGLLEVSVCQYNMCEYPHTICHPNLGNHFKELWHYDTGAVSCLYKRNFTYDVNATYLYFHFYQEGGTFYAYFTDTGFVTKFLFNVYLGMALSHYYVMPLTCIRRSKDGFNPEYWVTPLTPRQYLLAFNQDGIIFNAVDCMSDFMSEIKCKTQSIAPPTGVYELNGYTVQPIADVYRRKPDLPNCNIEAWLNDKSVPSPLNWERKTFSNCNFNMSSLMSFIQADSFTCNNIDAAKIYGMCFSSITIDKFAIPNRRKVDLQLGNLGYLQSSNYRIDTTATSCQLYYNLPAANVSVSRFNPSTWNKRFGFIEDSVFKPQPAGVLTDHDVVYAQHCFKAPKNFCPCSSCSGKNNGIGTCPAGTNSLTCDNLCTLDPITLKAPDTYKCPQSKSLVGIGEHCSGLAVKSDYCGNNSCTCQPQAFLGWSADSCLQGDKCNIFANFILHDVNNGLTCSTDLQKANTEIELGVCVNYDLYGISGQGIFVEVNATYYNSWQNLLYDSNGNLYGFRDYITNRTFMIHSCYSGRVSAAYHANSSEPALLFRNIKCNYVFNNSLTRQLQPINYSFDSYLGCVVNAYNSTAISVQTCDLTVGSGYCVDYFKNRGSASAITTGYRFTNFEPFTVNSVNDSLEPVGGLYEIQIPSEFTIGNMEEFIQTSSPKVTIDCAAFVCGDYAACKLQLVEYGSFCDNINAILTEVNELLDTTQLQVANSLMNGVTLSTKLKDGVNFNVDDINFAPVLGCLGSECSKASSRSAIEDLLFDKVKLSDVGFVEAYNNCTGGAEIRDLICVQSYKGIKVLPPLLSENQISGYTLAATSASLFPPWTAAAGVPFYLNVQYRINGLGVTMDVLSQNQKLIANAFNNALHAIQQGFDATNSALVKIQAVVNANSEALNNLLQQLSNRFGAISASLQEILSRLDPPEAEAQIDRLINGRLTALNAYVSQQLSDSTLVKFSAAQAMEKVNECVKSQSSRINFCGNGNHIISLVQNAPYGLYFIHFNYVPTKYVTAKVSPGLCIAGNRGIAPKSGYFVNVNNTWMYTGSGYYYPEPITENNVVVMSTCAVNYTKAPYVMLNTSIPNLPDFKEELDQWFKNQTSVAPDLSLDYINVTFLDLQVEMNRLQEAIKVLNHSYINLKDIGTYEYGSGYIPEAPRDGQAYVRKDGEWVLLSTFLGGLNDIFEAQKIEWHEHHHHHH |
| HKU1 A S protein | MLLIIFILPTTLAVIGDFNCTNFAINHKNTTVPRISEYVVDVSYGLGTYYILDRVYLNTTILFTGYFPKAGANFRDLSLKGTTYLSTLWYQKPFLSDFNNGIFSRVKNTKLYVNKTLYSEFSTIVIGSVFINNSYTIVVQPHNGVLDITACQYTMCEYPHTICKSKGSYRNESWHFDKSEPLCLFKKNFTYNVSTDWLYFHFYQERGTFYAYYADSGMPTTFLFSLYLGTLLSHYYVLPLTCNAISSNTDNETLQYWVTPLSKRQYLLKFDNRGVITNAVDCSSSFFSEIQCKTKSLLPNTGVYDLSGFTVKPVATVHRRIPDLPDCDIDKWLNNFNVPSPLNWERKIFSNCNFNLSTLLRLVHTDSFSCNNFDESKIYGSCFKSIILDKFAIPNSRRSDLQLGSSGFLQSSNYKIDTTSSSCQLYYSLPAINVTINNYNPSSWNRRYGFNNFNLSSHSVVYSRYCFSVNNTFCPCANPSFASSCKSHKPPSASCPIGTNYRSCESTTVLDRTDWCRCSCLPDPITAYDPRSCSQKKSLVGVGEHCAGFGVDEEKCGVLDGSYNASCLCSTDAFLGWSYDTCVSNNRCNIFSNFILNGINSGTTCSNDLLQPNTEVFTDVCVDYDLYGITGQGIFKEVSAVYYNSWQNLLYDSNGNIIGFKDFVTNKTYNIFPCYAGRVSAAFHQNASSLALLYRNLKCSYVLNNISLTTQPYFDSYLGCVFNADNLTDYSVSSCALRMGSGFCVDYNSPSSSSSRGSASSISASYRFVTFEPFNVSFVNDSIESVGGLYEIKIPTNFTIVGQEEFIQTNSPKVTIDCSLFVCSNYAACHDLLSEYGTFCDNINSILDEVNGLLDTTQLHVADTLMQGVTLSSNLNTNLHFDVDNINFKSLVGCLGPHCGSSSRSFFEDLLFDKVKLSDVGFVEAYNNCTGGSEIRDLLCVQSFNGIKVLPPILSESQISGYTTAATVAAMFPPWSAAAGIPFSLNVQYRINGLGVTMDVLNKNQKLIATAFNNALLSIQNGFSATNSALAKIQSVVNSNAQALNSLLQQLFNKFGAISSSLQEILSRLDPPEAQVQIDRLINGRLTALNAYVSQQLSDISLVKFGAALAMEKVNECVKSQSPRINFCGNGNHILSLVQNAPYGLLFMHFSYKPISFKTVLVSPGLCISGDVGIAPKQGYFIKHNDHWMFTGSSYYYPEPISDKNVVFMNTCSVNFTKAPLVYLNHSVPKLSDFESELSHWFKNQTSIAPNLTLNLHTINATFLDLYYEMNLIQESIKSLNNSYINLKDIGTYEGSGYIPEAPRDGQAYVRKDGEWVLLSTFLGGLNDIFEAQKIEWHEHHHHHH |
| HKU1 B S protein | MFLIIFILPTTLAVIGDFNCTNSFINHYNKTIPRISEDVVDVSLGLGTYYVLNRVYLNTSLLFTGYFPKSGANFRDLALKGSTYLSTLWYKPPFLSDFNNGIFSKVKNTKLYVNNTLYSEFSTIVIGSVFVNTSYTIVVQPHNGILEITACQYTMCEYPHTVCKSKGSIRNESWHIDSSEPLCLFKKNFTYNVSADWLYFHFYQERGVFYAYYADVGMPTTFLFSLYLGTILSHYYVMPLTCNAISPKIDNETLEYWVTPLSRRQYLLNFDEHGVITNAVDCSSSFLSEIQCKTQSFAPNTGVYDLSGFTVKPVATVYRRIPNLPDCDIDNWLNNVSVPSPLNWERRIFSNCNFNLSTLLRLVHVDSFSCNNLDKSKIFGSCFNSITVDKFAIPNRRRDDLQLGSSGFLQSSNYKIDISSSSCQLYYSLPLPNVTINNFNPSSWNRRYGFGSFNVSSYDVVYSDHCFSVNSDFCPCADPSVVNSCDKSKPLSAICPTGTKYRHCDLDTTLYVKNWCRCSCLPDPISTYSPNTCPQKKVVVGIGEHCPGLGINEEKCGTQLNHSSCSCSPDAFLGWSFDSCISNNRCNIFSNFIFNGINSGTTCSNDLLYSNTEVSTGVCVNYDLYGITGQGIFKEVSAAYYNNWQNLLYDSNGNIIGFKDFLTNKTYTILPCYSGRVSAAFYQNSSSPALLYRNLKCSYVLNNISFISQPFYFDSYLGCVLNAVNLTSYSVSSCDLRMGSGFCIDYALPSSRGSASGISSPYRFVTFEPFNVSFVNDSVETVGGLFEIQIPTNFTIAGHEEFIQTSSPKVTIDCSAFVCSNYAACHDLLSEYGTFCDNINNILNEVNDLLDITQLQVANALMQGVTLSSNVNTNLHSDVDNIDFKSLLGCLGSQCGSSSRSLLEDLLFNKVKLSDVGFVEAYNNCTGGSEIRDLLCVQSFNGIKVLPPILSETQISGYTTAATVAAMFPPWSAAAGVPFSLNVQYRINGLGVTMDVLNKNQKLIANAFNKALLSIQNGFTATNSALAKIQSVVNANAQALNSLLQQLFNKFGAISSSLQEILSRLDPPEAQVQIDRLINGRLTALNAYVSQQLSDITLIKAGASRAIEKVNECVKSQSPRINFCGNGNHILSLVQNAPYGLLFIHFSYKPTSFKTVLVSPGLCLSGDRGIAPKQGYFIKQNDSWMFTGSSYYYPEPISDKNVVFMNSCSVNFTKAPFIYLNNSIPNLSDFEAEFSLWFKNHTSIAPNLTFNSHINATFLDLYYEMNVIQESIKSLNSSFINLKEIGTYEGSGYIPEAPRDGQAYVRKDGEWVLLSTFLGGLNDIFEAQKIEWHEHHHHHH |
| HKU1 C S protein | MFLIIFILPTTLAVIGDFNCTNSFINHYNKTIPRISEDVVDVSLGLGTYYVLNRVYLNTTLLFTGYFPKSGANFRDLALKGSIYLSTLWYKPPFLSDFNNGIFSKVKNTKLYVNNTLYSEFSTIVIGSVFVNTSYTIVVQPHNGILEITACQYTMCEYPHTVCKSKGSIRNESWHIDSSEPLCLFKKNFTYNVSADWLYFHFYQERGVFYAYYADVGMPTTFLFSLYLGTILSHYYVMPLTCNAISSNTDNETLEYWVTPLSRRQYLLNFDEHGVITNAVDCSSSFLSEIQCKTQSFAPNTGVYDLSGFTVKPVATVYRRIPNLPDCDIDNWLNNVSVPSPLNWERRIFSNCNFNLSTLLRLVHVDSFSCNNLDKSKIFGSCFNSITVDKFAIPNRRRDDLQLGSSGFLQSSNYKIDISSSSCQLYYSLPLVNVTINNFNPSSWNRRYGFGSFNLSSYDVVYSDHCFSVNSDFCPCADPSVVNSCVKSKPPSAICPAGTKYRHCDLDTTLYVKNWCRCSCLPDPISTYSPNTCPQKKVVVGIGEHCPGLGINEEKCGTQLNHSSCFCSPDAFLGWSFDSCISNNRCNIFSNFIFNGINSGTTCSNDLLYSNTEISTGVCVNYDLYGITGQGIFKEVSAAYYNNWQNLLYDSNGNIIGFKDFLTNKTYTILPCYSGRVSAAFYQNSSSPALLYRNLKCSYVLNNISFISQPFYFDSYLGCVLNAVNLTSYSVSSCDLRMGSGFCIDYALPSSRGSASGISSPYRFVTFEPFNVSFVNDSVETVGGLFEIQIPTNFTIAGHEEFIQTSSPKVTIDCAAFVCSNYAACHDLLSEYGTFCDNINSILNEVNDLLDITQLQVANALMQGVTLSSNLNTNLHSDVDNIDFKSLLGCLGSQCGSSSRSLLEDLLFNKVKLSDVGFVEAYNNCTGGSEIRDLLCVQSFNGIKVLPPILSETQISGYTTAATVAAMFPPWSAAAGVPFSLNVQYRINGLGVTMDVLNKNQKLIANAFNKALLSIQNGFTATNSALAKIQSVVNANAQALNSLLQQLFNKFGAISSSLQEILSRLDPPEAQVQIDRLINGRLTALNAYVSQQLSDITLIKAGASRAIEKVNECVKSQSPRINFCGNGNHILSLVQNAPYGLLFIHFSYKPTSFKTVLVSPGLCLSGDRGIAPKQGYFIKQNDSWMFTGSSYYYPEPISDKNVVFMNSCSVNFTKAPFIYLNNSIPNLSDFEAEFSLWFKNHTSIAPNLTFNSHINATFLDLYYEMNVIQESIKSLNSSFINLKEIGTYEGSGYIPEAPRDGQAYVRKDGEWVLLSTFLGGLNDIFEAQKIEWHEHHHHHH |
